# The Frequency of Transfusion-Transmitted Infections in Healthy Blood Donors at King Abdulaziz Hospital, Makkah, Kingdom of Saudi Arabia

**DOI:** 10.3390/medicina61122153

**Published:** 2025-12-03

**Authors:** Saeed H. Halawani, Mohammad S. Aldosari, Fozeya S. Al-Zahrani, Muneera A. Bulushi, Roba S. Moamenah, Saeed F. Algufairy, Nada Bajuaifer, Yonis A. Allohibi, Amal Zaghloul

**Affiliations:** 1 Department of Hematology and Immunology, Faculty of Medicine, Umm Al-Qura University, Makkah 24381, Saudi Arabia; nabajuaifer@uqu.edu.sa (N.B.); n3-n4@hotmail.com (Y.A.A.); 2 Department of Clinical Pathology, King Abdulaziz Hospital, Makkah 24222, Saudi Arabia; aldosari_ms@yahoo.com (M.S.A.); manoooor1212@hotmail.com (M.A.B.); 3 Microbiology and Parasitology Department, King Abdulaziz Hospital, Makkah 24222, Saudi Arabia; foeyaa@moh.gov.sa (F.S.A.-Z.); rmoamenah@moh.gov.com (R.S.M.); 4 Microbiology and Parasitology Department, King Abdullah Medical City, Makkah 24246, Saudi Arabia; s.f.1410@hotmail.com; 5 Clinical Pathology Department, Faculty of Medicine, Ain Shams University, Cairo 11517, Egypt; amal_zaghlool@med.asu.edu.eg

**Keywords:** blood donors, transfusion-transmitted infections, TTIs, Kingdom of Saudi Arabia

## Abstract

*Background and Objectives*: Transfusion-transmitted infections (TTIs) impose a considerable healthcare burden globally. Despite rigorous screening protocols, these infections can still be present among apparently healthy blood donors, potentially compromising the safety of transfusion recipients. Understanding the frequency of TTIs among blood donors is crucial for ensuring a healthy blood supply and gaining insights into the epidemiology of these infections within a community. *Materials and Methods*: The main objective of this study is to determine the frequency of TTIs among healthy blood donors, aged 18 to 60 years, at King Abdulaziz Hospital in Makkah City, Saudi Arabia. Data was collected retrospectively at the blood bank center from 1 January 2023, to 31 December 2023. *Results*: There were 8831 blood donors included. Saudi participants emerged as the dominant nationality, comprising 57% of the total sample (5036 out of 8831 donors). The prevalence of TTIs among blood donors varied according to the individual markers used. The overall TTI reactivity rates were low. Anti-HBc was the most common TTI-positive marker (7.5%), followed by syphilis (0.5%), HBV NAT (0.3%), HBsAg, and anti-HCV (0.3%). On the other hand, the lowest TTI-positive markers were HIV-1/-P2 and HTLV-1/-2 (0.04%). In Saudi participants, the most prevalent TTI marker was anti-HBc with a rate of 5.8% (293 out of 5036), followed by HBsAg (0.3%), syphilis (0.3%), and HBV NAT (0.2%). *Conclusions*: The present study found that HBV outperformed other TTI markers compared to the regional reports. However, in our research and the earlier reports, the rates of seropositive patients were noticeably low for HIV, HTLV, and malaria, while the rate for syphilis was higher, particularly among non-Saudi donors. NAT assays are crucial for screening blood donations for TTIs, which can help the early detection of infections and significantly reduce serological window periods. For a precise estimation of the frequency of TTIs, large prospective multicenter studies from various regions of the KSA are required.

## 1. Introduction

Blood transfusion is essential for both medical and surgical practice in healthcare services. Transfusion-transmitted infections (TTIs) involve various fatal organisms such as hepatitis C virus (HCV), hepatitis B virus (HBV), human immunodeficiency virus (HIV), human T leukemia virus type 1 and type 2 (HTLV-1 and 2), and syphilis, which impose a substantial burden on health authorities globally [1]. Individuals with these infections may experience asymptomatic or latent progression before the presentation of clinical symptoms. Therefore, accurately estimating worldwide incidence rates is challenging [2]. Strict guidelines and measures are implemented globally to prevent the transmission of these infections. They continue to present among apparently healthy blood donors, which may compromise the safety of blood transfusions among recipients. Therefore, it is crucial to determine the frequency of TTIs among blood donors and study the epidemiology of these infections within a community.

In blood transfusions, TTIs persist as a significant concern in healthcare settings worldwide. The presence of TTIs among apparently healthy blood donors and the general population has been highlighted by numerous studies [3,4]. In 1995, Ahmad and co-authors reported that the prevalence of HBV among blood donors was remarkable [5]. In another study, Memish and coworkers found a notable frequency of HCV among blood donors in the region. These studies highlighted the need for comprehensive screening protocols [6].

In 2013, the WHO report on blood safety showed that the average rate of blood donation deferral worldwide was 12%, and in the Kingdom of Saudi Arabia (KSA), it was 6.5% [7]. In recent years, the KSA has made considerable progress in the services of blood transfusion by implementing strict guidelines to minimize the risk of TTIs [8]. However, TTIs continue as a potential threat to blood safety. Therefore, continuous surveillance efforts and supporting research are crucial to safeguard blood safety.

In the KSA, the City of Makkah faces a unique demographic and healthcare challenge. Determining the frequency of these infections among blood donors at King Abdulaziz Hospital is necessary to ensure the safety and efficacy of blood transfusion practices.

The primary objective of this study is to determine the frequency of TTIs among healthy blood donors at King Abdulaziz Hospital in Makkah, KSA. This work aims to study the prevalence of TTIs, including HIV, HCV, HBV, malaria, syphilis, and HTLV-1 and 2, among blood donors, which may contribute to a broader understanding of the epidemiology of TTIs, thereby facilitating evidence-based interventions to reduce the risk of TTIs.

The present study aims to expand available knowledge on the prevalence of TTIs in the KSA and examine the measures implemented to prevent the transmission of these infections. This may contribute to the ongoing efforts to ensure the safety of the blood supply in the KSA.

## 2. Materials and Methods

### 2.1. Study Design and Period

This study employs a cross-sectional design to investigate the prevalence of TTIs among blood donors at King Abdulaziz Hospital in Makkah City, Saudi Arabia. Data for this study were collected retrospectively from 1 January 2023 to 31 December 2023, a 12-month timeframe.

We applied the consecutive sampling technique to select the samples. The sample size was calculated using the Epi Info program from the CDC, version 3.0. The minimum sample size was calculated based on a 99.99% confidence interval and a prevalence of 6.5% [7], and 1% confidence limit of 6538. However, we increased our sample size to 9119 to avoid any dropouts due to missing information (Table S1). The final sample size obtained was 8831, still sufficient to apply a robust test of significance.

### 2.2. Ethical Consideration

Ethical approval was obtained from the Health Affairs’ General Directorate in Makkah City (IRB No. H-02-K-076-0123-869) and the Ethics Committee of King Abdulaziz Hospital.

### 2.3. Inclusion and Exclusion Criteria

The study includes records of all individuals, regardless of gender, aged 18 to 60 years, who donated blood during the specified study period and met the eligibility criteria for blood donation based on standard donor screening protocols. Records of individuals with incomplete or missing data necessary for assessing TTIs were excluded from the analysis.

The primary data sources include electronic medical records and laboratory databases, which were maintained in the information system of the blood bank at King Abdulaziz Hospital. The demographic and serological data of all blood donors were reviewed, and the data of eligible donors were selected and systematically extracted from the electronic database. Selected data were transferred to Excel spreadsheets (Microsoft Corp, Redmond, WA, USA) and were entered in a standardized format. Then, the data was organized and aligned as tables with anonymous numbers. All information was checked for duplicate entries and missing data, which were removed and carefully cleaned, and were eventually prepared for statistical analysis.

Blood samples were obtained from all blood donors and were screened for major TTIs, including HBV, HCV, HIV-1 and 2, malaria, syphilis, and HTLV-1 and 2. Serological and molecular investigations were used to screen for TTI. Chemiluminescent microparticle immunoassay (CMIA) was used to screen for hepatitis B surface antigen (HBsAg), hepatitis B core antibody (anti-HBc), HCV antibodies (anti-HCV), antigen/antibody combo HIV-1 and 2, anti-HTLV-1 and 2, and syphilis (The Abbott Alinity Immunology Analyzer) (Abbott Laboratories, Abbott Park, IL, USA) (sensitivity of 100% and specificity to over 99.73%). Screening for malaria was conducted using rapid diagnostic tests (RDTs) and microscopic examination of thick and thin blood smears. Serological tests, such as rapid plasma reagin (RPR) or treponemal antibody tests, were used for syphilis screening. Serologically false-negative or false-positive results can be excluded by using nucleic acid amplification technology (NAT). Therefore, NAT was performed in conjunction with serological screening tests to detect HBV, HCV, and HIV in all blood samples. The Procleix Panther System was used, which employs Transcription-Mediated Amplification (TMA) and Hybridization Protection Assay (HPA) to detect viral nucleic acids, aiming for earlier detection of infections and reducing the window period. (Procleix Panther System HBV/HCV/HIV Assay) (Transcription Mediated Amplification, TMA) (Grifols Diagnostic Solutions Inc., Barcelona, Spain). This assay can detect HIV-1 RNA, HIV-2 RNA, HCV RNA, and HBV DNA with a specificity of 100% and a sensitivity of greater than 95%. All samples were retested in duplicate to reject false-positive or false-negative results. All tests were conducted with established standard operating procedures (SOPs) and guidelines recommended by regulatory authorities and international health organizations. Quality control measures were implemented to ensure the accuracy and reliability of laboratory test results, including regular calibration of equipment, proficiency testing, and adherence to internal quality assurance protocols.

### 2.4. Data Analysis

Descriptive statistics, including frequencies, percentages, means, and standard deviations, were used to outline sociodemographic characteristics and the prevalence of TTIs among blood donors. Statistical analysis, using SPSS version 25 (IBM, SPSS Inc., Armonk, NY, USA), was performed to calculate prevalence rates, assess associations between variables, and identify potential risk factors for TTIs. The chi-square test of significance was applied to measure the association between demographic variables. Multivariate analysis was performed to determine the most significant factors and the potential confounders.

## 3. Results

### 3.1. Sociodemographic Characteristics

During the study period, 8832 individuals donated blood at King Abdulaziz Hospital. Due to missing or incomplete data, 1 was excluded, whereas 8831 were included in the study. The gender distribution within the study population was significantly skewed towards males, with 8516 participants identifying as male (96.4%) and only 315 individuals identifying as female (3.6%) (Table 1).

The descriptive results for the prevalence of TTI markers among Saudi donors showed that male donors accounted for 94.7% (4771 out of 5036) of the total donor population, and female donors comprised 5.3% (265 out of 5036). The gender distribution within the study population was evaluated. Four distinct age categories were assessed: 20 years or less, 21–35 years, 36–50 years, and 50 years or above. The majority of participants observed among the two middle-aged groups, 21 to 35 years and 36 to 50 years, were distributed at 57% and 32%, respectively.

Saudi participants emerged as the dominant nationality, comprising 57% of the total sample (5036 out of 8831 donors). Yemen, Egypt, and Afghanistan followed, contributing 11%, 6.6%, and 2.9%, respectively. Notably, some nationalities were represented by only a handful of individuals, suggesting the presence of smaller, less-represented communities within the study population.

### 3.2. Distribution Sero-Prevalence of Transfusion-Transmitted Infections Markers Among Donors

The study explored various TTI markers among blood donors. The prevalence of TTIs among blood donors varies according to the individual markers used. Anti-HBc was the most common TTI-positive marker (7.5%), followed by syphilis (0.5%), HBV NAT (0.3%), HBsAg (0.3%), and anti-HCV (0.3%) (Table 2). In Saudi participants, the most prevalent TTI marker was anti-HBc with a rate of 5.8% (293 out of 5036), followed by HBsAg (0.3%) and syphilis (0.3%). On the other hand, the lowest TTI-positive markers were HIV-1/-P2 and HTLV-1/-2 (0.04%) among all blood donors. None of the blood donors showed malaria infection, indicating a prevalence rate of 0% (0 cases out of 8831 donors) (Table 2).

- Hepatitis B virus

Anti-HBc (Hepatitis B Core Antibody) screening identified 662 cases of reactive results, accounting for a prevalence rate of 7.5%. However, HBsAg (Hepatitis B Surface Antigen) findings exhibited 25 cases of reactive results, corresponding to a prevalence rate of 0.3% (25 out of 8831 donors) (Table 2). In Saudi participants, anti-HBc positive results were observed in 5.8% (293 out of 5036) of the donors. In addition, 13 (0.3%) of the Saudi donors had HBsAg-positive results (Table 3).

Hepatitis B virus nucleic acid amplification technology (HBV NAT) testing revealed 28 cases of reactive results among all donors, corresponding to a prevalence rate of 0.3% (28 out of 8831 donors) (Table 2). Out of the 28 HBV NAT-positive results, 2 were females. Among Saudi donors, HBV NAT-positive results were found in 0.2% (12 out of 5036) of donors (Table 3).

- Syphilis

The rapid plasma reagin (RPR) screening for syphilis infection identified 41 cases of reactive results, corresponding to a prevalence rate of 0.5% (41 out of 8831 donors). RPR-positive results were found in 15 Saudi blood donors (0.3%).

- Hepatitis C virus

Anti-HCV (Hepatitis C Virus Antibody) testing detected 24 cases of reactive results, indicating a prevalence rate of 0.3% (24 out of 8831 donors). Regarding HCV NAT, only 3 (0.03%) out of all donors had reactive results (Table 2). Among Saudi donors, only 7 (0.1%) were found to have HCV-positive results, and none of them showed reactivity with HCV NAT (Table 3).

- HTLV and HIV

HTLV-1 and HTLV-2 tests identified 4 cases of reactive results, accounting for a prevalence rate of 0.04% (4 out of 8831 donors). A substantial proportion of donors (99.96%) were negative for both HTLV-1 and HTLV-2 (Table 2). In Saudi blood donors, HTLV-1 and -2 revealed that one donor tested positive (0.02%), with the vast majority testing negative (99.98%) (Table 3).

HIV antigen serological screening for HIV and HIV P24 antigen revealed 4 cases of reactive results, indicating a prevalence rate of 0.04% (4 out of 8831 donors). Most donors (99.96%) were negative for HIV and HIV P24 antigen (Table 2). HIV NAT was reactive in 3 donors (0.03%). For Saudi blood donors, only 1 male donor tested positive for HIV and HIV P2 (0.02%). However, this Saudi blood donor had a non-reactive result for HIV NAT (Table 3).

### 3.3. Comparison Between Saudi and Non-Saudi Donors Across Age, Gender Distribution, and Infection Screening Results

This study highlights statistically significant differences between Saudi and non-Saudi blood donors, particularly in gender distribution and certain infection markers. The comparison between Saudi and non-Saudi blood donors showed that both are heavily dominated. However, non-Saudi donors showed higher male predominance (98.7%) compared to Saudi donors (94.7%) (*p* < 0.001) (Table 4). The analysis of age-group distribution in relation to nationality demonstrates statistically significant differences between Saudi and non-Saudi participants. There is a pattern in which the extremes of the age spectrum, the youngest and oldest groups, are more heavily represented by Saudis, whereas the middle-aged ranges show a more even distribution (Table 4). The study revealed a highly significant association between age group and nationality (*p* < 0.001) (Table 4).

Table 4 describes the association of demographic and clinical variables with nationality. There were statistically significant differences between age group, gender, HBV NAT, syphilis, anti-HCV, anti-HBc, and nationality with *p* value <0.001, <0.001, <0.001, <0.001, <0.044, <0.007, <0.005, and <0.001, respectively. However, other variables show no significant difference (Table 4).

The overall prevalence of TTIs is low in both groups. The non-Saudi donors show higher reactivity rates for most infections, notably HBV markers, HCV, and syphilis. The study found that anti-HBc showed the largest disparity between the two groups (9.7% vs. 5.8%) (*p* < 0.001) (Table 3). Moreover, syphilis reactivity showed a significant difference between Saudi (0.3%) and non-Saudi (0.7%) donors (*p* < 0.05). The difference between the groups was also statistically significant for HBV NAT, and anti-HCV (*p* < 0.05 for each marker) (Table 3). There were no reported cases of malaria infections in either group.

### 3.4. Association Between Independent Demographic Variables and the Clinical Outcome Variables

This study aimed to identify potential confounders influencing the association between demographic factors (age group, gender, and nationality) and various serological and molecular screening markers, including anti-HBc, syphilis, HBV NAT, and anti-HCV. The multivariate regression analysis provided important insights into how demographic characteristics shape the distribution of infectious disease markers within the studied population. The researcher employed the multiple linear regression analysis at a 95% confidence interval. The independent variables were age group, gender, and nationality, while the dependent variables were anti-HBc, syphilis, HBV NAT, and anti-HCV. The variables HBs Ag, HCV NAT, HTLV-1 and 2; HIV and HIVP2; and HIV NAT were not included in the model as they showed no significant association in the cross-tab.

The analysis showed a good model fit: F (38827) = 132.67, *p* < 0.001, Adj R^2^ = 0.043, and change R^2^ = 0.043 for anti-HBc. The analysis revealed that nationality has a negative impact on anti-HBc (β = −0.08, t = −7.54, *p* < 0.001. Furthermore, the age group has a negative effect on anti-HBc (β = −0.19, t = −18.52, and *p* < 0.001. The two potential confounders identified for anti-HBc are nationality and age group. In addition, the analysis showed model fit: F (38827) = 2.547, *p* = 0.054, Adj R^2^ = 0.001, and change R^2^ = 0.001 for syphilis. There is no significant association between syphilis and the independent variables. Therefore, there is no potential confounder identified for syphilis.

For HBV NAT, the analysis showed model fit: F (38827) = 2.753, *p* = 0.041, Adj R^2^ = 0.001, and change R^2^ = 0.001. The nationality has a negative effect on HBV NAT (β = −0.02, t = −2.091, *p* = 0.037. The potential confounder identified for HBV NAT is nationality. Moreover, the analysis showed a model fit: F (38827) = 2.748, *p* = 0.041, Adj R^2^ = 0.001, and change R^2^ = 0.001 for anti-HCV. The analysis shows that nationality has a negative effect on anti-HCV (β = −0.03, t = −2.657, *p* = 0.008. The potential confounder identified for anti-HCV is nationality.

In summary, nationality was identified as a potential confounder for anti-HBc, HBV NAT, and anti-HCV, while age group was a potential confounder for anti-HBc. No potential confounders were found for syphilis (Table 5).

## 4. Discussion

Transfusion-transmitted infections (TTIs) are a group of infections that raise concern due to their silent presence and progression. Unfortunately, their presence was detected in healthy-looking donors, despite considerable efforts to improve screening protocols. Thus, TTIs continue to pose a potential risk to the safety of blood collected from healthy-looking donors. These infections can compromise the safety of transfusion recipients. The present study was conducted to identify the frequency of TTIs among blood donors at King Abdulaziz Hospital in Makkah City, Saudi Arabia. The prevalence rates of TTI markers among blood donors provide critical insights into the potential risk of TTIs within the donor population.

The current study indicates notable demographic and epidemiological differences between Saudi and non-Saudi blood donors. Saudi participants emerged as the dominant nationality, comprising 57% of the total sample (5036 out of 8831 donors), reflecting the study’s likely geographical focus or recruitment strategy. Additionally, it was found that 96.4% of the blood donors were male and 3.6% were female. The study found that both Saudi and non-Saudi groups are heavily male-dominated. However, non-Saudi participants showed higher male predominance (98%) compared to Saudi donors (94%). Likewise, Altayar et al. reported that 96.9% of blood donors were males and 3.1% were females [9]. In 2018, a study by Alaidarous and colleagues found that 97.6% of the donors were males and 2.4% were females [10]. This notable gender disparity may be attributed to behavioral and sociocultural factors. Moreover, females are diagnosed earlier during prenatal care. In addition, this can be expected as male donors were always considered more regular long-term blood donors than females, especially since females were at risk of developing iron deficiency anemia (IDA) at several phases of their lives (such as early adulthood, child-bearing age) more than males [11].

The analysis of age-group distribution in relation to nationality demonstrates varying proportional representation of Saudi and non-Saudi individuals. The overall distribution revealed that the mid-adult population participated more in blood donation than other age categories. The findings of the present study show a more balanced representation among nationalities in the mid-adult population. These patterns may reflect underlying demographic structures, health-seeking behaviors, and recruitment characteristics. Studying these age-related differences is essential for planning health strategies, resource allocation, and future research involving diverse population groups.

Our study investigated the frequency of TTIs among blood donors over 12 months (1 January to 31 December 2023), which varies according to the individual tests used. All infections are rare, with the majority being ˂1%. Anti-HBc was the most common TTI-positive marker (7.5%), followed by syphilis (0.5%), HBV NAT (0.3%), HBsAg, and anti-HCV (0.3%). The present study found that the frequency of seropositive results for TTIs with serological markers can reach up to 7.5%, and 0.3% when NAT tests were used. Among Saudi donors, the prevalence rate of TTIs decreased from 7.5% to 5.8% with serology and decreased from 0.3% to 0.2% with NAT tests. This reduction in the prevalence rate from the multicultural population donors to Saudi donors only suggests behavioral and socio-cultural factors contributing to the occurrence of the infection.

The seroprevalence of TTIs among blood donors has been evaluated in various provinces of the KSA. At the King Faisal Specialist Hospital in Makkah, KSA, Altayar et al. found that the 7-year cumulative prevalence of TTIs among blood donors was 7.93% [9]. In 2024, Minshawi and coworkers reported the prevalence of TTIs in Makkah, KSA as 7.4% [12]. However, only serological tests were performed, and confirmatory tests were not specified. In a retrospective study by Albshri and coworkers (2024) in Makkah, KSA, it was found that the prevalence of positive TTIs was 13.5% of the blood donors, but the rates of TTI reactivity among Saudi and non-Saudi donors were not reported [13]. In another research in Najran province of the KSA, Alshehri and colleagues found positive TTI markers in 10.9% of blood donors [14]. Serological tests were used, while the NAT tests were not applied. These reports are comparable to our results. On the other hand, Kabrah and coauthors recruited 5473 blood donors at the Security Force Hospital (SFH) in Makkah, KSA, from January 2015 to December 2018. They found that TTI markers were positive in 14.3% of the donors [15]. However, a study conducted in the Central region of KSA by Alabdulmonem et al. revealed that the prevalence of TTIs (1%) was lower than that of the present study (9%) [16]. However, only serological tests were performed, and confirmatory tests were not specified.

Our results and those reported at different hospitals in the KSA were lower than those published in other parts of Asia and Africa [9]. They reported a frequency rate of 18.7% in New Guinea, 37.4% in Mozambique, and 24% in Burkina Faso [11,17,18]. The lower frequency reported by our research group than in other countries in Asia and Africa can be justified by the comparatively low prevalence of these infections among the population of the Makkah region compared to those countries [19,20,21]. Furthermore, this can be due to the strong guidelines designed to decrease the risk of transmission of these infections between individuals.

In this study, HBV infection was the most prevalent TTI among blood donors. Anti-HBc was the most frequent TTI-positive marker (7.5%) among all blood donors. In Saudi participants, anti-HBc-positive results were observed in 5.8% (293 out of 5036) of the donors, and 13 (0.3%) had HBsAg-positive results. Non-Saudi donors have consistently higher rates, especially for HBV and syphilis markers. The present study suggests that anti-HBc positivity is influenced by nationality-based and age-related population characteristics. The higher infection reactivity rates, particularly for anti-HBc, suggest more historical exposures or health backgrounds to HBV. It has been reported that HBV was the most observed infection in blood transfusion centers in the KSA [22]. Our results are comparable to those reported by other research groups in KSA [12,16,23,24]. However, these reports showed a lower prevalence of hepatitis B among blood donors in comparison to that found in Sub-Saharan African countries. This could be referred to as low socioeconomic conditions in those countries.

In 2015, Elbjeirami et al. reported that anti-HBc among donors in Makkah, KSA, was 6.7% [25]. In another study by Alshehri and researchers, the most prevalent biomarker among TTI-positive blood donors was anti-HBc (52.5%), followed by syphilis (5.5%), anti-HCV (4.6%), anti-HTLV I/II (1.6%), and anti-HIV-1/-2 (0.8%) [14]. Minshawi and colleagues reported that HBV was the most prevalent infection among the donors, with 6.1% positivity for anti-HBc antibodies and 0.4% for HBsAg [12].

The rapid plasma reagin (RPR) tests identified 41 cases of reactive results, corresponding to a prevalence rate of 0.5% (41 out of 8831 donors). A vast number of donors (99.5%) tested negative for VDRL. VDRL-positive results were found in 15 Saudi blood donors (0.3%), while the majority tested negative (99.7%). Our results are comparable to those reported by other research groups in KSA. A study by Alaidarous and coauthors reported that 16 (0.5%) cases reacted to Treponema Pallidum (TP) antibodies (TPHA) [10]. In the KSA, another study by Wanni et al. found that 54 cases (0.45%) had seropositive results for syphilis, and the prevalence rate of syphilis among Saudis was 0.36% [26]. They also found that out of the 54 positive cases, 28 (51.9%) were non-Saudi workers who were screened for employment.

The prevalence of HCV markers was comparatively low among all blood donors. Anti-HCV (Hepatitis C Virus Antibody) testing detected 24 cases of reactive results, indicating a prevalence rate of 0.3% (24 out of 8831 donors). When HCV NAT was used, only 3 out of the 24 donors had reactive results (0.03%). Among Saudi donors, only 7 (0.1%) were found to have HCV-positive results, and none of them showed reactivity with HCV NAT. The frequency of HCV among blood donors reported in this study is comparable with national reports in the KSA. In Makkah City, KSA, a recent retrospective study by Minshawi and colleagues reported that the overall prevalence of HCV-positive cases was 0.4% [12]. Similarly, a study in Makkah, KSA, reported the seroprevalence of HCV as 0.44% [25]. The low prevalence rate of seropositive HCV reported in the KSA can be attributed to the strict measures for screening overseas workers before traveling to the KSA and the national premarital screening program enforced by the Saudi health authorities.

HTLV-1, HTLV-2, and HIV serological screening tests identified 4 cases for each disease, accounting for a prevalence rate of 0.04% (4 out of 8831 donors). The study found that HIV NAT was reactive in 3 blood donors (0.03%). In the Saudi blood donors, screening tests for HTLV-1, -2, and HIV revealed that one male donor tested positive (0.02%), with the vast majority testing negative (99.98%). However, this Saudi blood donor had a non-reactive result for HIV NAT. The present study is comparable with many earlier reports from Saudi Arabia for HTLV and HIV infections, and the prevalence of these infections was very low. In a study in Makkah, a prevalence rate of 0.07% of all blood donors had HIV reactivity [26]. Alaidarous and researchers identified a prevalence rate of 0.06% for HIV infection among blood donors [10]. In 2024, Minshawi et al. reported that the prevalence rate of seropositive donors was 0.06% for HIV, 0.13% for HTLV, and 0.34% for syphilis [12]. However, the HIV NAT test was not utilized. Our study and the earlier reports from the KSA revealed that syphilis, HIV, and HTLV infections are very low among blood donors. These results also showed that infections with syphilis are common among non-Saudi donors.

Recently, NATs for HCV, HIV, and HBV have been used in blood screening. After infections, there is a window period from the initial infection to the detection of antibodies against the pathogen. NAT assays can detect genetic materials even before the appearance of antibodies. The traditional serological screening tests for TTIs require the presence of these antibodies.

Albshri and coworkers found that the relationship between NAT-HBV and HBsAg was strong (*p* < 0.001) [13]. In addition, Kabrah et al. reported that serological results were 57 (1.07%) HBsAg, 292 (5.34%) HBsAb, 388 (7.1%) HBcAb, 13 (0.24%) HCV, and 5 (0.09%) HIV. The rates of TTIs declined when NAT tests were used. The results for NAT HBV, HCV, and HIV showed that 50 (0.91%), 1 (0.0002%), and 3 (0.05%) were reactive, respectively [15]. The present study found that serological assays revealed 25 (0.3%) HBsAg, 662 (7.5%) HBcAb, 24 (0.3%) anti-HCV, and 4 (0.04%) HIV. The results of HBV NAT, HCV, and HIV NAT were reduced to 28 (0.3%), 3 (0.03%), and 3 (0.03%), respectively. It can be concluded from these results that NAT tests can help in the early detection of infections and reduce serological window periods. Consequently, NAT assays should be used in conjunction with standard serological tests to exclude false-negative blood results.

Across several outcome variables, nationality emerged as the most consistent confounder. Significant negative associations were found between nationality and anti-HBc, anti-HCV, and HBV NAT. This pattern suggests that certain national groups differ in exposure history, vaccination coverage, or underlying epidemiological risk patterns for hepatitis viruses. For anti-HBc, the association was strong, indicating that differences in past HBV exposure or immunological memory may vary substantially between nationalities. Similar but smaller effects were seen for anti-HCV and HBV NAT, where nationality likely reflects disparities in healthcare access, screening practices, cultural behaviors, or historical infection prevalence in the home country. The consistent role of nationality highlights the need for population-specific prevention strategies. Public health programs may benefit from tailoring awareness campaigns, vaccination initiatives, and screening protocols to subgroups with differing risk profiles.

Age group was identified as a significant confounder only for anti-HBc. The negative association suggests that younger individuals have lower exposure to hepatitis B infection or a higher likelihood of being vaccinated under more recent immunization programs. This finding aligns with epidemiological data indicating declining HBV exposure rates in younger populations, especially in regions with strengthened vaccination policies. Gender was not shown as a significant predictor for any of the outcome variables. This suggests that, within this population, serological and NAT-based screening markers do not differ substantially between males and females. This could reflect uniform exposure patterns or effective gender-neutral public health interventions.

Both syphilis and HIV NAT showed no significant associations with any demographic variables. For syphilis, the lack of association may indicate that syphilis exposure in this population is sporadic and not clustered within specific demographic groups. Similarly, the absence of demographic effects on HIV NAT may reflect low prevalence, successful prevention strategies, or homogenous distribution across groups.

These findings provide insight into the prevalence of TTI markers among blood donors in Saudi Arabia, highlighting the occurrence of specific infections within the donor population. A major strength of this analysis is the large sample size (*n* = 8831), which increases the precision of estimates and reliability of findings. However, the relatively low adjusted R^2^ values in some models indicate that demographic factors explain only a small proportion of the variance in clinical outcomes. This suggests that other unmeasured factors, such as behavioral risk, socioeconomic status, vaccination history, or prior medical interventions, may play important roles. Moreover, this study is a single-center study with gender disparity; therefore, the findings cannot be generalized.

## 5. Conclusions

Health authorities face enormous challenges and burdens from the impact of TTIs on healthcare systems. This retrospective single-center study was conducted at King Abdulaziz Hospital, Makkah City, the western region of Saudi Arabia, to determine the frequency of TTIs among blood donors. The frequency of TTIs in our study was extremely low, which may be attributed to various factors such as the implementation of strict screening measures for donor selection, the national premarital screening program, enforcement of strict measures to prevent the transmission of infections, and strict guidelines for screening overseas workers before traveling to the KSA.

The present study found a notable gender disparity with fewer women participating in blood donations. This highlights the need for targeted awareness initiatives to improve women’s engagement. The rates of seropositive patients with HBV were higher than those of other TTI markers compared to regional reports. Therefore, efforts to decrease this rate should be encouraged by intensifying community awareness campaigns and emphasizing the importance of vaccination to reduce the risk of transmission. In our research and earlier reports, the rates of seropositive patients were noticeably low for HIV, HTLV, and malaria. In addition, it was found that HBV and syphilis were higher among non-Saudi donors. Therefore, an enhanced screening program, continuous monitoring, and targeted health education may achieve efficiency by focusing on demographics with high prevalence rates.

NAT assays are crucial in screening blood donations for TTIs, which can help the early detection of infections and significantly reduce serological periods. Therefore, NAT assays should be used in conjunction with standard serological tests to exclude false-negative blood results.

Identifying confounders is essential for understanding disease distribution and designing effective health interventions. The finding that nationality and age group significantly influence viral hepatitis markers underscores the value of demographic-aware public health planning. Clinics and blood banks may consider enhanced screening or education programs for specific national groups or age cohorts. Finally, for a precise estimation of the frequency of TTIs, large prospective multicenter studies from various regions of the KSA are required.

## Figures and Tables

**Table 1:** Study sample characteristics for blood donors (*n* = 8831).

Variable	Response	Number	%
Age	≤20	389	4.4
	21–35	5045	57.1
	36–50	2865	32.4
	≥50	532	6.1
	Total	8831	100
Gender	Male	8516	96.4
	Female	315	3.6
	Total	8831	100
Nationality	Saudi	5036	57
	Non-Saudi	3795	43
	Total	8831	100

**Table 2:** Distribution seroprevalence of transfusion-transmitted infections markers among all donors (*n* = 8831).

TTI Markers	Reactivity *n* (%)	
	Reactive	Negative
ANTI-HBc	662 (7.5)	8169 (92.5)
SYPHILIS	41 (0.5)	8790 (99.5)
HBV NAT	28 (0.3)	8803 (99.7)
HBsAg	25 (0.3)	8806 (99.7)
ANTI-HCV	24 (0.3)	8807 (99.7)
HTLV-1 and -2	4 (0.04)	8827 (99.96)
HIV and HIVP2	4 (0.04)	8827 (99.96)
HIV NAT	3 (0.03)	8828 (99.97)
HCV NAT	3 (0.03)	8828 (99.97)
MALARIA	0 (0)	8831 (100)

**Table 3:** Association of transfusion-transmitted infection markers with nationality between Saudi (*n* = 5036) and non-Saudi donors (*n* = 3795).

TTI Markers	Saudis Reactivity *n* (%)		Non-Saudi Reactivity *n* (%)		*p* Value
	Reactive	Negative	Reactive	Negative	
ANTI-HBc	293 (5.8)	4743 (94.2)	369 (9.7)	3426 (90.3)	<0.001
SYPHILIS	15 (0.3)	5021 (99.7)	26 (0.7)	3769 (99.3)	0.007
HBV NAT	12 (0.2)	5024 (99.8)	16 (0.4)	3779 (99.6)	0.044
HBsAg	13 (0.3)	5023 (99.7)	12 (0.3)	3783 (99.7)	0.377
ANTI-HCV	7 (0.1)	5029 (99.9)	17 (0.4)	3778 (99.6)	0.005
HTLV-1 and -2	1 (0.02)	5035 (99.98)	3 (0.1)	3792 (99.9)	0.215
HIV and HIVP2	1 (0.02)	5035 (99.98)	3 (0.1)	3792 (99.9)	0.215
HIV NAT	0 (0.0)	5036 (100)	3 (0.2)	3792 (99.8)	0.079
HCV NAT	0 (0.0)	5036 (100)	3 (0.2)	3792 (99.8)	0.079

**Table 4:** Association of demographic variables with nationality between Saudi (*n* = 5036) and non-Saudi donors (*n* = 3795).

Variable	Response	Saudis *n* (%)	Non-Saudi *n* (%)	*p* Value
Age	≤20	295 (5.9)	94 (2.5)	˂0.001
	21–35	2740 (54.4)	2305 (61)	
	36–50	1582 (31.4)	1283 (34)	
	≥50	419 (8.3)	113 (3)	
	Total	5036	3795	
Gender	Male	4771 (95)	3745 (98.7)	˂0.001
	Female	265 (5)	50 (1.3)	
	Total	5036	3795	

**Table 5:** Association of transfusion-transmitted infection markers with age, gender, and nationality.

TTI Markers		Age	Gender	Nationality
ANTI-HBc Adjusted R^2^ = 0.043, R^2^ change = 0.043, F change = 132.67, *p* ˂ 0.001	β	−0.193	0.14	−0.079
	t	−18.52	1.289	−7.539
	*p* value	<0.001	0.198	<0.001
SYPHILIS Adjusted R^2^ = 0.001, R^2^ change = 0.001, F change = 2.547, *p* = 0.054	β	−0.003	−0.008	−0.029
	t	−0.269	−0.751	−2.722
	*p* value	0.788	0.453	0.006
HBV NAT Adjusted R^2^ = 0.001, R^2^ change = 0.001, F change = 2.753, *p* = 0.041	β	−0.019	−0.014	−0.022
	t	−1.772	−1.324	−2.091
	*p* value	0.076	0.186	0.037
ANTI-HCV Adjusted R^2^ = 0.001, R^2^ change = 0.001, F change = 2.748, *p* = 0.041	β	0.005	0.007	−0.028
	t	0.431	0.679	−2.657
	*p* value	0.666	0.497	0.008

## Data Availability

The data supporting this study’s findings are available from the corresponding author upon request.

## Supplementary Materials

The following supporting information can be downloaded at: https://www.mdpi.com/article/10.3390/medicina61122153/s1, Table S1: Sample Size for Frequency in a Population.

## Abbreviations

The following abbreviations are used in this manuscript:

TTIs	Transfusion-transmitted infections
KSA	Kingdom of Saudi Arabia

## References

[1] World Health Organization (2021). Global Progress Report on HIV, Viral Hepatitis and Sexually Transmitted Infections.

[2] Attaullah S., Khan S., Khan J. (2012). Trend of transfusion transmitted infections frequency in blood donors: Provide a road map for its prevention and control. J. Transl. Med..

[3] Aabdien M., Selim N., Himatt S., Hmissi S., Merenkov Z., AlKubaisi N., Abdel-Rahman M.E., Abdelmola A., Khelfa S., Farag E. (2022). Prevalence and trends of transfusion transmissible infections among blood donors in the State of Qatar, 2013–2017. BMC Infect. Dis..

[4] Alharazi T., Alzubiery T.K., Alcantara J.C., Qanash H., Bazaid A.S., Altayar M.A., Aldarhami A. (2022). Prevalence of Transfusion-Transmitted Infections (HCV, HIV, Syphilis and Malaria) in Blood Donors: A Large-Scale Cross-Sectional Study. Pathogens.

[5] Ahmad M.S., Mahtab A.M., Abdullatif A.S., Tashkandy M.A., Kashreed M.S., Maulana A. (1995). Prevalence of antibodies against the hepatitis C virus among voluntary blood donors at a makkah hospital. Saudi J. Kidney Dis. Transpl..

[6] Memish Z.A., Knawy B.A., El-Saed A. (2010). Incidence trends of viral hepatitis A, B, and C seropositivity over eight years of surveillance in Saudi Arabia. Int. J. Infect. Dis..

[7] World Health Organization (WHO) Global Status Report on Blood Safety and Availability.

[8] Alkhamis A. (2012). Health care system in Saudi Arabia: An overview. East. Mediterr. Health J..

[9] Altayar M.A., Jalal M.M., Kabrah A., Qashqari F.S.I., Jalal N.A., Faidah H., Baghdadi M.A., Kabrah S. (2022). Prevalence and Association of Transfusion Transmitted Infections with ABO and Rh Blood Groups among Blood Donors in the Western Region of Saudi Arabia: A 7-Year Retrospective Analysis. Medicina.

[10] Alaidarous M., Choudhary R.K., Waly M.I., Mir S., Bin Dukhyil A., Banawas S.S., Alshehri B.M. (2018). The prevalence of transfusion-transmitted infections and nucleic acid testing among blood donors in Majmaah, Saudi Arabia. J. Infect. Public Health.

[11] Anees M., Jawad A., Hashmi I. (2007). Distribution of ABO and Rh blood group alleles in Mandi Bahauddin district of Punjab, Pakistan. Proc. Pak. Acad. Sci..

[12] Minshawi F., Abdulshakoor A.A., Alwakil E.M., Basfar G.T., Kabrah S., Aslam A., Almasmoum H., Mujalli A., Moaminah R.H., Almoalad G.A. (2024). Seroprevalence of transfusion-transmitted infections among blood donors in Makkah, Saudi Arabia. J. Infect. Dev. Ctries..

[13] Albshri M.H., Ghoth A.A., Mutwalli A.A., Alzahrani N.S., Bahattab S.S., Hanbali B.A., Althaqafi M.H., Alqarni A.M., Alsulimani M.M., Alzahrani N.K. (2024). The Prevalence of Transfusion-Transmitted Infections and Nucleic Acid Testing Among Blood Donors in Makkah, Saudi Arabia. Clin. Lab..

[14] Alshehri A.A., Adebayo Irekeola A., Merae Alshahrani M., Mohammed Abdul K.S., Ahmed Asiri S., Aboluluy B.F., Abdullah Al Awadh A., Hassan Alhasaniah A., Abdullah Almazni I., Alshamrani S.A. (2024). Serological markers of transfusion transmissible infections and ABO blood groups in Najran, Saudi Arabia. Saudi Med. J..

[15] Kabrah S.M., Alandijany T.A., Felimban R.I., Alserihi R.F., Theyab A., Ebid G.T. (2023). The Prevalence of Transfusion-Transmitted Infection Markers among Blood Donors at Saudi Hospital, Makkah. Clin. Lab..

[16] Alabdulmonem W., Shariq A., Alqossayir F., AbaAlkhail F.M., Al-Musallam A.Y., Alzaaqi F.O., Aloqla A.A., Alodhaylah S.A., Alsugayyir A.H., Aldoubiab R.K. (2020). Sero-prevalence ABO and Rh blood groups and their associated Transfusion-Transmissible Infections among Blood Donors in the Central Region of Saudi Arabia. J. Infect. Public Health.

[17] Chavhan A. (2011). Allelic Frequency of ABO And Rh D Blood Group Among The Banjara Caste Population of Akola District, Maharashtra, India. Nat. Prec..

[18] Handoo S., Bala S.S. (2013). Distribution of ABO and Rhesus Blood Groups in Kashmir Valley. Int. J. Sci. Res..

[19] Gao X., Cui Q., Shi X., Su J., Peng Z., Chen X., Lei N., Ding K., Wang L., Yu R. (2011). Prevalence and trend of hepatitis C virus infection among blood donors in Chinese mainland: A systematic review and meta-analysis. BMC Infect. Dis..

[20] Belkacemi M., Merbouh M.A. (2023). Seroprevalence of Human Immunodeficiency Virus, Hepatitis C Virus, and Hepatitis B Virus Among Blood Donors in Sidi Bel Abbes, West Algeria. Cureus.

[21] Shiferaw E., Tadilo W., Melkie I., Shiferaw M. (2019). Sero-prevalence and trends of transfusion-transmissible infections among blood donors at Bahir Dar district blood bank, northwest Ethiopia: A four year retrospective study. PLoS ONE.

[22] Alzahrani F.M., Shaikh S.S., Alomar A.I., Acharya S., Elhadi N. (2019). Prevalence of Hepatitis B Virus (HBV) Among Blood Donors in Eastern Saudi Arabia: Results From a Five-Year Retrospective Study of HBV Seromarkers. Ann. Lab. Med..

[23] Alsughayyir J., Almalki Y., Alburayk I., Alalshaik M., Aljoni I., Kandel M., Alfhili M.A., Alabdullateef A.A. (2022). Prevalence of transfusion-transmitted infections in Saudi Arabia blood donors: A nationwide, cross-sectional study. Saudi Med. J..

[24] Alshahrani M.Y., Alamri A.M., Alshahrani A.S., Alshehri A.A., Alsulaiman A.S., Alshahrani A.J., Dabaan M.A., Aldhbaan S.T., Moosa R.A., Abdullah M. (2021). Prevalence of selected blood-borne infectious diseases among voluntary blood donors in abha, Saudi Arabia. Southeast Asian J. Trop. Med. Public Health.

[25] Elbjeirami W.M., Arsheed N.M., Al-Jedani H.M., Elnagdi N., Abou Eisha H.M., Abdulwahab A., Abdulateef N.A.B., Hezam E., Al-Allaf F.A. (2015). Prevalence and trends of common transfusion transmitted infections using serological and nucleic acid markers in Saudi Blood Donors. J. Blood Disord. Transfus..

[26] Wanni N.H.O., Dossary R.A., Obeid O.E., Qahtani N.H.A., Siddiqui Z.I., El-Badry A.A., Alkharsah K.R. (2021). Seropositivity of syphilis among individuals screened in a tertiary hospital in the Eastern Province of Saudi Arabia. Ann. Saudi Med..

