# Supplementary figures and images for: The Frequency of Transfusion-Transmitted Infections in Healthy Blood Donors at King Abdulaziz Hospital, Makkah, Kingdom of Saudi Arabia
Source: Medicina (Kaunas). 2025 Dec 3;61(12):2153. doi: 10.3390/medicina61122153 (PMC12735232; doi:10.3390/medicina61122153)

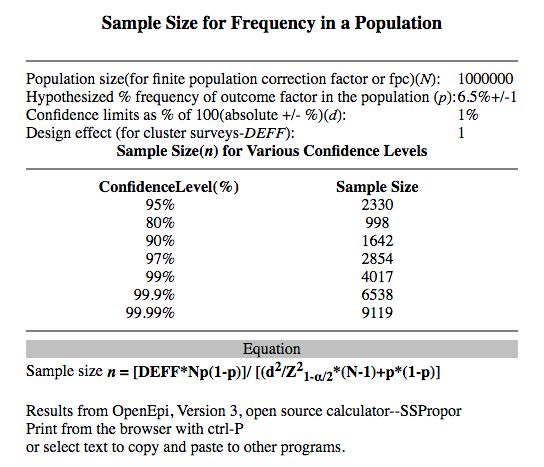

Supplement: Supplementary file 1 [file medicina-61-02153-s001.zip › medicina-3883400-supplementary.png]
